# Waiting list mortality and the potential of donation after circulatory death heart transplantations in the Netherlands

**DOI:** 10.1007/s12471-020-01505-y

**Published:** 2020-11-06

**Authors:** S. Roest, S. E. Kaffka genaamd Dengler, V. van Suylen, N. P. van der Kaaij, K. Damman, L. W. van Laake, J. A. Bekkers, M. Dalinghaus, M. E. Erasmus, O. C. Manintveld

**Affiliations:** 1 Department of Cardiology, Thorax Centre, Erasmus MC, University Medical Centre Rotterdam, Rotterdam, The Netherlands; 2 Department of Cardiothoracic Surgery, University Medical Centre Utrecht, Utrecht, The Netherlands; 3 Department of Cardiothoracic Surgery, University Medical Centre Groningen, Groningen, The Netherlands; 4 Department of Cardiology, University Medical Centre Groningen, Groningen, The Netherlands; 5 Department of Cardiology, University Medical Centre Utrecht, Utrecht, The Netherlands; 6 Department of Cardiothoracic Surgery, Thorax Centre, Erasmus MC, University Medical Centre Rotterdam, Rotterdam, The Netherlands; 7 Department of Paediatrics, Division of Paediatric Cardiology, Erasmus MC, University Medical Centre Rotterdam, Rotterdam, The Netherlands

**Keywords:** Donation after circulatory death, Heart transplantation, Waiting list, Mortality

## Abstract

**Background:**

With more patients qualifying for heart transplantation (HT) and fewer hearts being transplanted, it is vital to look for other options. To date, only organs from brain-dead donors have been used for HT in the Netherlands. We investigated waiting list mortality in all Dutch HT centres and the potential of donation after circulatory death (DCD) HT in the Netherlands.

**Methods:**

Two different cohorts were evaluated. One cohort was defined as patients who were newly listed or were already on the waiting list for HT between January 2013 and December 2017. Follow-up continued until September 2018 and waiting list mortality was calculated. A second cohort of all DCD donors in the Netherlands (lung, liver, kidney and pancreas) between January 2013 and December 2017 was used to calculate the potential of DCD HT.

**Results:**

Out of 395 patients on the waiting list for HT, 196 (50%) received transplants after a median waiting time of 2.6 years. In total, 15% died while on the waiting list before a suitable donor heart became available. We identified 1006 DCD donors. After applying exclusion criteria and an age limit of 50 years, 122 potential heart donors remained. This number increased to 220 when the age limit was extended to 57 years.

**Conclusion:**

Waiting list mortality in the Netherlands is high. HT using organs from DCD donors has great potential in the Netherlands and could lead to a reduction in waiting list mortality. Cardiac screening will eventually determine the true potential.

## What’s new?

- One out of seven adult patients and one out of four paediatric patients die while on the waiting list before a donor heart becomes available.
- Every second patient was not transplanted while on the waiting list between 2013 and 2017.
- Donation after circulatory death (DCD) heart transplantation (HT) has great potential to increase the number of heart transplants performed in the Netherlands.
- Cardiac screening in DCD donors is essential for the success of a Dutch DCD HT programme.

## Introduction

The shortage of suitable donor hearts worldwide has led to changes in the accepted donor pool. While the average donor age was 29 years before 2000, this has now increased to 43 and is still rising [1]. Besides the use of older donor hearts, the complexity of the patients on the waiting list has also increased, with many more patients receiving mechanical circulatory support; waiting times have also increased. Nevertheless, the 10-year cumulative survival has significantly improved from between 53 and 65% for patients receiving transplants before 2000 to between 68 and 76% since 2000 [1–3]. However, the number of patients on the waiting list has risen due to the introduction of left ventricular assist devices (LVADs) [4]. Up to now, only hearts donated after brain death (DBD) have been used for heart transplantation (HT) in the Netherlands. Since the successful introduction of machine perfusion, interest in the transplantation of hearts donated after circulatory death (DCD) has increased significantly [5]. In 2017, the Papworth group reported an increase of almost 30% in the number of transplants since the introduction of the DCD procedure [6]. Recently, similar findings were demonstrated in Australia, showing an increase of almost 22% [7]. In the Netherlands, DCD transplantations are already commonly performed for liver, lung, kidney and pancreas with good results and represent around 50% of all transplant procedures [8]. Despite this advantage, the question remains what the potential is for DCD HT in the Netherlands and what the impact could be on waiting list mortality as well as on time on the waiting list.

In this study we investigated the waiting list mortality in all Dutch HT centres to illustrate the urgency of implementing DCD HT, and studied the potential of DCD HT in the Netherlands.

## Methods

In order to calculate waiting list mortality, a cohort was evaluated consisting of patients who were already on the waiting list or were newly listed for HT in the Netherlands between January 2013 and December 2017. Data were retrospectively collected by chart review. For these patients, date of listing, date of heart failure diagnosis and blood type were noted. Follow-up was continued until September 2018. At the end of the follow-up period, the status of the patient was categorised into six groups: heart transplant, death while on the waiting list, removed from the waiting list due to improved cardiac condition, removed from the waiting list due to deterioration of the condition, removed from the waiting list due to a different reason, and still listed.

A second cohort was used to calculate the potential of DCD HT in the Netherlands. This cohort was obtained from the Dutch Transplant Society and consisted of all donors who donated their organs for DCD transplantation (lung, liver, kidney or pancreas) in the Netherlands between January 2013 and December 2017. All of these patients were screened retrospectively for contraindications for DCD HT. Contraindications were scored according to the Papworth list of contraindications as published by Messer et al. [6]. In addition to the inclusion criteria of Messer et al., both DCD type III donors (controlled DCD, donors awaiting cardiac arrest) and DCD type V donors (controlled DCD, donation after euthanasia) were included [9], as donation after euthanasia is allowed in the Netherlands [10]. All inclusion and exclusion criteria are listed in Tab. 1. For the final analyses, two age limits were used: a maximum donor age of 50 years (donor age limit at the start of the DCD HT programme in Papworth) and a maximum donor age of 57 years (as is currently used by the English and Australian groups following successful introduction of their DCD HT programme).

**Table 1:** Inclusion and exclusion criteria used for eligibility testing of donation after circulatory death (*DCD*) donors

Inclusion criteria	Exclusion criteria
Maastricht classification III or V DCD donor	Cardiac abnormalities
Age ≤57 years	– History of cardiac surgery
Ejection fraction >50% (if known)	– History of coronary abnormalities
No abnormalities on echocardiography (if known)	– History of myocardial infarction
	– History of congenital heart disease
	Malignancies
	– Malignancies in the past five years
	– History of malignant melanoma
	– Primary intracerebral lymphoma
	– Secondary intracerebral malignancies
	Infections
	– Human immunodeficiency virus
	– Hepatitis B/C positive
	– Tuberculosis
	Insulin-dependent diabetes
	Supportive therapy
	– Noradrenaline >0.3 µg/kg per min
	– Adrenaline
	– Dobutamine
	– Dopamine
	Functional warm ischaemia time >30 min (if known)^a^
	Other exclusion criteria as used in donation after brain death

^a^Functional warm ischaemia time starts when the systolic blood pressure drops under 50 mm Hg and ends when cardioplegia is administered

The study was performed in accordance with the Declaration of Helsinki and the Medical Research Involving Human Subjects Act (non-WMO research).

### Statistical analysis

Categorical variables are presented as an absolute number with percentages (%). Continuous variables are presented as mean ± standard deviation when normally distributed or as median with interquartile range (IQR) when not normally distributed.

Continuous variables between groups were compared with a Student *t*-test when normally distributed or a Mann-Whitney test with non-normally distributed data. When more than two groups were included, one-way ANOVA (normally distributed) or Kruskal-Wallis test (not normally distributed) were used.

For categorical variables the chi-square test was used or Fisher’s exact test when appropriate.

Survival analysis and comparisons in the number of HTs per blood type were performed with Kaplan-Meier methods. Kaplan-Meier event curves were compared using the log-rank test.

Statistical analyses were performed using SPSS, version 25.0 (SPSS Inc., IBM, Chicago, IL, USA) and GraphPad Prism version 5.0a (GraphPad Software, La Jolla, CA, USA).

## Results

### Waiting list mortality

Between January 2013 and December 2017, a total of 395 patients were on the HT waiting list. Their baseline characteristics are summarised in Tab. 2. The median age at the time of listing was 50 years (IQR 40–58), with 35 (9%) being under the age of 18. The minority were female, 146 (37%) patients. Blood types O (48%) and A (42%) were most common (Tab. 2). The median time between heart failure diagnosis and listing was 5.2 (1.7–10.2) years. The median duration between heart failure diagnosis and listing was significantly shorter in patients < 18 years old compared with adults (1.3 vs 5.8 years, respectively, *p* = 0.012).

**Table 2:** Baseline characteristics of patients on the waiting list between 2013 and 2017

Baseline characteristics	All (*n* = 395)	Adults (*n* = 360)	Children (*n* = 35)
Age at listing	50 (40–58)	51 (43–59)	8 (2–13)
Female gender	146 (37)	123 (34)	23 (66)
Blood type			
– A	164 (42)	154 (43)	10 (29)
– B	32 (8)	27 (8)	5 (14)
– AB	9 (2)	8 (2)	1 (3)
– O	190 (48)	171 (48)	19 (54)
Time from HF diagnosis to listing (years)	5.2 (1.7–10.2)	5.8 (2.2–10.7)	1.3 (0.2–3.5)

Baseline characteristics with categorical variables are presented in absolute numbers (percentages) and continuous variables with medians (IQR)

*HF* heart failure

At the end of follow-up, 196 (50%) patients had undergone a HT and 28 (7%) were removed from the waiting list (see Tab. 3). Nine (2%) were removed because of deterioration of the condition and 11 (3%) because of improvement of their cardiac condition. Eight (2%) were removed due to another reason. In total, 60 (15%) patients died while on the waiting list before a suitable donor heart became available. Nine (26%) paediatric patients died versus 51 (14%) adult patients (*p* = 0.069).

**Table 3:** Endpoint at the end of follow-up

	All (*n* = 395)	Adults (*n* = 360)	Children (*n* = 35)
Transplant	196 (50)	172 (48)	24 (69)
Died on waiting list	60 (15)	51 (14)	9 (26)
Removed from waiting list due to:			
– Deteriorated condition	9 (2)	9 (3)	0 (0)
– Improved cardiac function	11 (3)	11 (3)	0 (0)
– Other	8 (2)	7 (2)	1 (3)
Still on waiting list	111 (28)	110 (31)	1 (3)

Endpoints are given with categorical variables in absolute numbers (%)

**Fig. 1:**
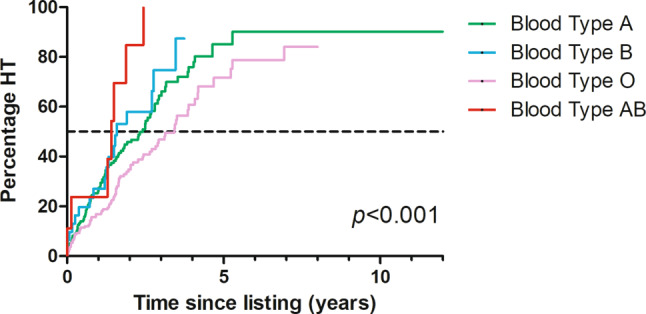
The number of patients receiving a heart transplant (*HT*) since being added to the waiting list, stratified by blood type

The median waiting time for patients until HT was 2.6 years (IQR 2.2–2.9). Patients listed under the age of 18 who underwent a transplantation had a significantly shorter median waiting time compared to adults (0.5 years vs 2.8 years, respectively, *p* < 0.001). Maximum time on the waiting list for a patient who received a transplant was 6.9 years. Overall, the maximum time on the waiting list was 8.0 years. This specific patient died while on the waiting list. Waiting time for HT was significantly different between blood types (median of 3.4 vs 2.4 vs 1.5 vs 1.4 years for blood types O, A, B and AB, respectively, *p* < 0.001; see Fig. 1). The total percentage of patients who eventually underwent a transplantation during this period also differed between blood types. Patients with blood type AB received a transplant most often, followed by blood types B, A and O (78% vs 59% vs 55% vs 42%, respectively, *p* = 0.01).

### Potential for DCD heart transplantation

**Fig. 2:**
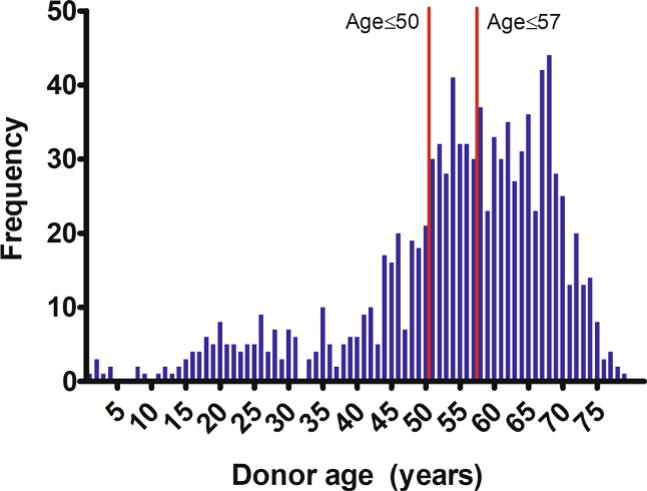
Age distribution of donation after circulatory death donors in the Netherlands between 2013 and 2017

Between January 2013 and December 2017, 1006 DCD procedures were performed in the Netherlands. The average donor age was 53.4 ± 14.8 years; 319 were aged ≤50 and 551 were ≤57 years (Fig. 2).

Most donors (959, 95%) were DCD type III (controlled DCD, donor awaiting cardiac arrest), 35 (4%) were DCD type V (controlled DCD, donation after euthanasia), 10 (1%) were DCD type II (uncontrolled DCD, unsuccessful resuscitation) and 2 (0%) were DCD type I (uncontrolled DCD, dead in the out-of-hospital setting).

**Fig. 3:**
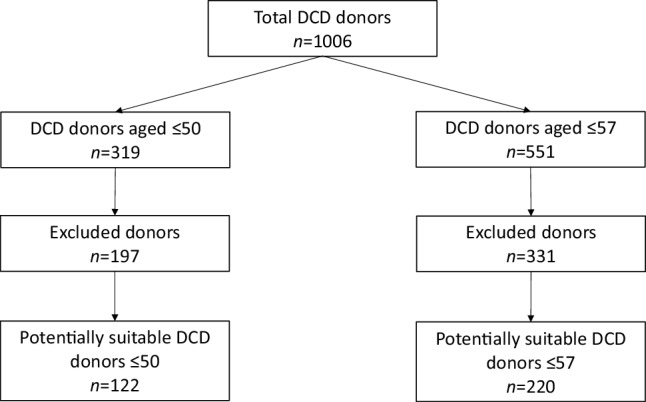
Potential for transplantation of hearts from donation after circulatory death (*DCD*) donors aged under 50 or 57 years

In Fig. 3, the potential for donors aged ≤50 or ≤57 years is displayed. When applying a maximum donor age of 50 years and the exclusion criteria mentioned in Tab. 1, 122 donors could have been suitable candidates for heart donation. When the maximum donor age of 57 years was applied, this number increased to 220 potential donor hearts. Hypothetically, this would mean an increase of over 100% in comparison to the number of DBD transplants performed between 2013 and 2017 (*n* = 184). This would only be the case if all of these donors had hearts suitable for donation, died within the maximal functional warm ischemia time (FWIT) and had matching recipients listed. Furthermore, cardiac screening was performed in only 1% of the cases.

The median age of the donors who were eligible for DCD HT (≤50 years old) was 43 years (IQR 27–47) When the age limit of donors was set at 57 years, the median donor age was 49.5 years (IQR 40–54). Twelve donors were under the age of 18, the youngest being 7 years old. Three of these paediatric donors had a body weight <25 kg, which is the lowest weight recorded for the successful use of machine perfusion in DCD HT to date (no reference given/anecdotal evidence only).

## Discussion

In this study, we found a substantial waiting list time for HT, which was accompanied by a high waiting list mortality in the Netherlands (15%). Furthermore, we established great potential of DCD HT.

In the Netherlands, the number of HTs has remained stable, but very low (~40/year) in the past few years [8]. Yet, the number of patients on the waiting list has greatly increased. As such, there is reason to explore the potential of DCD donors. The present study suggests that at least part of the donor heart shortage could be alleviated by extending HT to DCD donations. Of course, the actual potential depends on cardiac screening of DCD donors as well as consent for heart donation. In addition, the actual number of DCD HTs will be dependent on several other factors. For example, a potential donor must have a minimum weight (and thus age), as at least 1 l of donor blood is needed to prime the machine perfusion device that is used during DCD HT [6, 7]. At present, no standard cardiac screening (electrocardiography, echocardiography and coronary status) is performed in DCD donors. Cardiac screening will determine how many donors are eventually suitable to donate their heart for DCD HT. It is anticipated that some hearts will not be deemed suitable for HT because of coronary artery disease and/or left ventricular dysfunction. Finally, the duration of FWIT is of importance. FWIT starts when the systolic blood pressure drops below 50 mm Hg and ends when cardioplegia is administered (with an upper limit of 30 min), effectively stopping ischaemia [6].

### DCD heart transplantation in other countries

In several countries, the potential of DCD HT has been calculated, varying between 4 and 17% in the USA and Belgium and 56% in the UK [11–15]. Australia and the UK started DCD HT in 2014 and 2015, respectively. Since the start of the DCD HT programmes, both countries have seen a significant increase in HT numbers of 22 and 33%, respectively [6, 7]. The potential in the Netherlands could be even higher. DCD donation is already accepted for the kidney, liver, lung and pancreas, which could make the implementation of DCD HT more accessible [8]. Furthermore, donation after euthanasia is possible in the Netherlands [10], which could increase the number of heart donors even more. We believe that DCD HT (compared to other countries) could increase the number of HTs by 10–15 annually, increasing the number of HTs by 26–40% over the coming years.

### Using machine perfusion for DCD heart transplantation

Cardiac screening before life support of the donor is withdrawn in the intensive care unit is a must to assess which hearts might meet the inclusion criteria. Hearts from DCD donors can suffer from significant ischaemic injury prior to organ procurement [16], which is one of the factors leading to primary graft dysfunction [17]. With the introduction of machine perfusion in transplantation, it is possible to perfuse a potential donor heart ex vivo until it is transplanted in the recipient. Theoretically, this may reduce the cardiac ischaemia/reperfusion injury [16]. One of the disadvantages of DCD HT is that the cardiothoracic surgeon cannot functionally assess the heart function in a loaded condition. When the heart has been explanted and is beating on the machine perfusion device, surrogate markers such as lactate are used to assess the quality of the heart [6]. When lactate levels rise during machine perfusion, the heart might be rejected for HT. When lactate levels show a downward trend, the heart is deemed suitable for transplantation [5]. However, it is questionable whether lactate is the ideal biomarker. The Australian group started with a level of 5 mmol/l for lactate as the threshold for DCD HT. However, HT was also successfully performed in patients with a lactate concentration >5 mmol/l. This is why the group decided to use a downward trend instead of a threshold of 5 mmol/l for lactate [7].

Alternative markers need to be explored in order to obtain more solid evidence to decide whether a heart is suitable for HT. Donor management, organ resuscitation, preservation and evaluation are essential to minimise the risk of primary graft dysfunction and successful further application of DCD heart transplantation in the future.

### Waiting list mortality

Adult mortality is comparable with that in the UNOS database (a US heart transplant database), 14% versus 16%, respectively [18]. It is agreed that all children under the age of 16 are listed as International High Urgent, while donors in the Eurotransplant zone are prioritised for paediatric HT patients [19]. This status is maintained until a child turns 16 years of age, after which the child is put on the adult waiting list (except when the skeletal age is still under the age of 16). DCD HT could help reduce mortality.

Of course, timely referral for HT is essential. An acronym to determine which patients should be referred to a tertiary centre for LVAD and/or HT evaluation is ‘I NEED HELP’ (Tab. 4; [20]). LVADs can keep patients alive and in good condition while on the waiting list. Yet patients with severe right ventricular failure or congenital cardiac defects are rarely candidates for LVAD implantation. Furthermore, complications during LVAD therapy often become refractory in the long term. As such, the donor pool needs to be expanded to decrease waiting time. Whether DCD HT will decrease waiting time will depend on several factors, including the actual number of donors screened and deemed suitable for DCD HT as well as the evolution of LVAD therapy over time.

**Table 4:** ‘I NEED HELP’ acronym

*I*	*I*notropes	Previous or ongoing requirement for dobutamine, milrinone, dopamine or levosimandan
*N*	*N*YHA classification/*N*atriuretic peptides	Persisting NYHA class III or IV and/or persisting high BNP or NT-proBNP
*E*	*E*nd-organ dysfunction	Worsening renal or liver dysfunction in the setting of heart failure
*E*	*E*jection fraction	Very low ejection fraction <20%
*D*	*D*efibrillation shocks	Recurrent appropriate defibrillator shocks
*H*	*H*ospitalisations	More than 1 hospitalisation for heart failure in the last 12 months
*E*	*E*dema/*E*scalating diuretics	Persisting fluid overload and/or increasing diuretic requirement
*L*	*L*ow blood pressure	Consistently low BP with a systolic <90–100 mm Hg
*P*	*P*rognostic medication	Inability to up-titrate (or need to decrease or cease) ACEI, β‑blockers, ARNIs or MRAs

(Adapted from [20] with permission)

*ACEI* angiotensin-converting enzyme inhibitor, *ARNI* angiotensin-receptor neprilysin inhibitor, *BNP* B-type natriuretic peptide, *BP* blood pressure, *MRA* mineralocorticoid receptor antagonist, *NT-proBNP* N-terminal pro-b-type natriuretic peptide, *NYHA* New York Heart Association

### Donor pool expansion

Up until this year, an opt-in system was used in the Netherlands, where people have to actively register to be a donor [21]. As of mid-2020 this has been changed to an opt-out system, potentially increasing the available donor pool.

Although long-term results of DCD HT are not known yet, mid-term (5-year) outcomes are at least comparable to those of DBD donation [6, 7]. To decrease time on the waiting list as well as waiting list mortality, we strongly believe that DCD HT should be introduced in the Netherlands in all HT centres to expand the donor pool. We expect this to become a reality in 2021.

In conclusion, mortality rates on the waiting list are high in the Netherlands, in part due to a shortage of donors. DCD HT has great potential in the Netherlands to decrease time on the waiting list for HT and as such also to reduce waiting list mortality. Cardiac screening of DCD donors will be essential to determine the true potential of DCD HT in the Netherlands.
